# T Cell Aging: A Review of the Transcriptional Changes Determined from Genome-Wide Analysis

**DOI:** 10.3389/fimmu.2013.00121

**Published:** 2013-05-20

**Authors:** Guobing Chen, Ana Lustig, Nan-ping Weng

**Affiliations:** ^1^Laboratory of Molecular Biology and Immunology, National Institute on Aging, National Institutes of HealthBaltimore, MD, USA

**Keywords:** aging, thymocytes, T cells, naïve and memory T cells, CD28^−^ T cells

## Abstract

Age carries a detrimental impact on T cell function. In the past decade, analyses of the genome-scale transcriptional changes of T cells during aging have yielded a large amount of data and provided a global view of gene expression changes in T cells from aged hosts as well as subsets of T cells accumulated with age. Here, we aim to review the changes of gene expression in thymocytes and peripheral mature T cells, as well as the subsets of T cells accumulated with age, and discuss the gene networks and signaling pathways that are altered with aging in T cells. We also discuss future direction for furthering the understanding of the molecular basis of gene expression alterations in aged T cells, which could potentially provide opportunities for gene-based clinical interventions.

## Introduction

Age carries a detrimental impact on T cell function. Age-associated decline of T cell function is complex and occurs at multiple levels (Haynes and Swain, 2006). Alteration in transcription is arguably one of the most studied and identifiable age-associated change in T cells. As part of the adaptation to a changing microenvironment with age (Linton et al., 2005), T cells undergo substantial changes in gene expression, resulting in enhanced or reduced aspects of their functions (Pawelec et al., 2005; Weng, 2006). Reported age-associated alterations in T cell functions include: increased cytotoxicity, enhanced secretion of inflammatory cytokines, reduced antigen-induced proliferation, and a decrease in cell division. The precise details of age-associated changes in transcription are beginning to be revealed, but the functional significance of these transcriptional alterations and their contribution to the age-associated changes in T cell function have not been fully characterized. A better understanding of the transcriptional changes in T cells that are associated with aging is an essential first step toward further functional assessment and eventual clinical interventions for older adults.

The development of methods, such as microarrays, allow the genome-scale assessment of gene expression and provide a means to understand the scope and magnitude of gene expression changes during aging (Prolla, 2005). The current commercially available whole genome arrays (Agilent, Affymetrix, and Illumina) contain the information of over 30,000 transcripts with most of the annotated protein-coding genes of human and mouse, and readily assess RNAs isolated from a few thousand to millions of cells. With sufficient biological repeats and proper statistical tools (Hyatt et al., 2006), the global gene expression pattern and significantly enhanced or reduced expressed genes are promptly identified; this provides sufficient transcriptional details to match the complexity of the age-associated changes. However, connecting the individual altered expressed genes to the altered functional networks and signaling pathways with aging is a monumental task.

In the past a few years, studies of global gene expression in T cells with aging have evolved from analyzing whole organ/tissue/whole populations of T cells to comparing well-defined subsets of T cells from different lymphoid tissues or organs, and from identification of significantly altered genes to connecting the gene networks and signaling pathways that are associated with these altered genes. The cumulative evidence shows that age-related alterations of transcription in T cells can be found in thymocytes (Lustig et al., 2007, 2009; Griffith et al., 2012), in peripheral mature T cells (Remondini et al., 2010), and T cell subsets (Fann et al., 2005; Mirza et al., 2011). Although neither the causes nor the consequences of all the transcriptional changes in T cells with age are currently known, the identification and validation of these age-associated changes pave a new avenue for a better understanding of cellular and functional alterations associated with aging. This information will be essential for developing strategies to slow down or even reverse the course of T cell aging. The purpose of this review is to summarize the age-associated gene expression changes on a global level that have been observed in recent studies of T cells and their subsets. We also discuss a potential link between the transcriptional changes and functional changes in aged T cells.

## Transcriptional Changes in Aged Thymus and Peripheral Blood T Cells

### Thymocytes

Initial microarray experiments were focused on comparing gene expression of the total organ/tissue/T cells between young and old hosts (mouse and human). Global gene expression analysis using total thymus from mice revealed that the largest number of gene expression changes with age, within total thymus, occurred in the categories of transcription regulators and enzymes, and were mostly involved with cell growth, proliferation, and death (Lustig et al., 2007). One of the more notable transcription regulators that decreased expression with age was the glucocorticoid receptor, which has been implicated in T cell development and survival within the thymus. Also detected was an increased expression of several heat shock proteins, which are necessary for cell survival.

A subsequent study focused specifically on thymocytes, demonstrated age-associated changes in the expression of genes involved in TCR signaling, antigen presentation, and lymphocyte development and function (Lustig et al., 2009). These age-related changes included increased expression of CXCR4 and CXCR6, as well as a decrease in CCL25. Another key change was an increase in Vav1, which is important for T cell development and activation. Genes involved with cell function included changes in various ribosomal proteins, which could indicate changes in translational activity with age, and a decrease in S100A, a calcium-binding protein involved in various cell functions. Interestingly, increased gene expression was consistent in genes associated with cancer.

An unexpected and significant difference was found in gene expression analyses of the immunoglobulin gene family from the thymus of old and young mice (Lustig et al., 2007). This observation has been noted in various gene expression studies (Schumacher et al., 2008; Swindell, 2009), even though some of these studies focused specifically on thymocytes or splenic T cells. Further analysis revealed the apparent presence of immunoglobulin on the cell surfaces of thymocytes as well as splenic T cells, but not intracellularly, indicating this might be an autoreactive phenomenon occurring with age (Lustig et al., 2009).

Not surprisingly, the age-associated change of the environment in which each T cell is located has an effect on the gene expression profile of thymocytes. Griffith et al. (2012) demonstrated that the most profound age-associated changes in gene expression (up to 30% of the genes) were found in the thymic stroma, and such changes affected gene expression changes in all subsets of thymocytes, which only had expression changes in about 3–4% of their total genes. However, the small proportion of changes within the developing thymocytes could be the most critical ones involved in T cell aging.

### Mature T cells in the periphery

Changes in gene expression with age, specifically within the murine spleen, mirrored the overall trends of other organs; these included an increase in immune response, stress, and apoptosis related genes, along with a decrease in genes involved in cell growth, energy utilization, and lipid and carbohydrate metabolism (Schumacher et al., 2008). An interesting observation was that many of these expression patterns were similar between mouse strains with shortened, normal, or longer life spans, but the changes occurred at a pace relative to the expected life span for each strain.

Sole focus on gene expression analyses of T cells purified from mouse spleens showed critical gene expression changes in the old mice that may be responsible for their alterations in T cell function (Han et al., 2006). The authors found that old T cells express higher *Socs3* and lower growth factor independent 1 (*Gfi-1*) compared to young T cells, which could contribute to the age-associated decline in proliferation. They also found the increase of apoptosis in old T cells could be explained by higher *Gadd45* and lower *Bcl2* in old T cells compared to young T cells. In addition, the authors observed an age-associated increase in many immunoglobulin-associated genes, as well as genes associated with innate immunity, such as lysozyme M and myeloperoxidase; moreover, this study showed a decrease in TCR-associated genes such as TCR-beta and various isoforms of *Cd3*, indicating that both the innate and adaptive arms of the immune system are involved in aging trends. Other categories of genes affected included structural proteins, calcium-binding proteins, and genes involved in electron transport.

Three basic patterns of changes in gene expression emerged from a study of T cells in peripheral blood of various aged adults: first, some genes increased in middle age then decreased; second, genes that decreased in middle age then increased, and lastly, some genes decreased with age (Remondini et al., 2010). Interestingly, they found no group of genes that only increased with age. Global clustering analysis revealed that the gene expression pattern in “successfully aged” adults (older than 90 years) more closely matched the patterns of the younger groups. Most of the genes involved in T cell signaling were in the group which increased in middle age, then decreased. Although it is difficult to strictly compare the patterns between the human and mouse studies with age, some common genes are evident. Not surprisingly, these genes are associated with TCR signaling, cytokines and their receptors, and cancer-related genes. Other common genes between human and mouse that changed expression with age included those from more general signaling pathways, such as Jak/Stat, PPAR, and mTOR signaling. This agrees with the observation that changes in gene expression occurring within aging T cells mirror changes occurring in other cell types throughout the body.

Genome-wide analyses of transcription alterations identified the change associated with aging as a whole of either tissue or of total T cells. As lymphoid tissue consists of different types of cells (T and B cells, stromal cells, etc.), total T cells also consist of different subsets (naïve, effector, and memory), it is unknown whether the identified age-associated changes are a result of aging in all types of cells or in a specific type of cell or a subset of a cell type. Therefore, it is necessary to further analyze the gene expression changes with age in defined a defined cell type, such as T cells, and their subsets.

## Gene Expression Changes of T Cell Subsets in Aged Subjects

### Human CD4 T cells

Although a global comparison of gene expression in naïve CD4 T cells with age is not available, a study comparing global gene expression of memory CD4 T cells (CD45RA^−^) between young (20–30 years old) and old (70–75 years old) human subjects show that the overall gene expression profiles of CD4 memory T cells are similar (Czesnikiewicz-Guzik et al., 2008). Based on the fold change (> or <1.25), 536 genes were identified as age-related, including up-regulated genes such as HLA region genes, *CCR4*, *CCR8*, *CD26*, *CD58*, *IL17Rb*, and *LAIT1*, and down-regulated genes such as *CD28*, *CCR6*, *KLRB1*, and *KLRC2*.

Another recent study compared gene expression of CD4 T cells between young and old donors after *in vitro* stimulation (Bektas et al., 2013). This study focused on examining changes in TCR-inducible gene expression via the NF-κB pathway, and found that most NF-κB target genes are not induced in a sustained manner in CD4 T cells from older compared to younger donors. Those genes that failed to exhibit sustained expression include *CXCL10*, *TNFAIP2*, *CCL2*, and *CXCL5*. On the other hand, a subset of NF-κB target genes such as *CXCL1*, *CXCL11*, *IL1a*, and *IL6*, continue to be up-regulated even in the absence of NF-κB induction. *IL1* and *IL6* are associated with a chronic pro-inflammatory state in the elderly and are dysregulated in CD4 T cells from old donors. In addition, the authors identified some immune function-related genes that were highly induced after 2 h of stimulation in old donors, including cell surface receptor and signaling molecules (*SAMD4A*, *CD83*, *KCTD12*, *SOCS1*, and *CTLA4*) and effector molecules (*GZMH*). Interestingly, some of the most noteworthy age-associated changes in murine models, such as up-regulation of the chemokines CCL3 and CCL4 and the cytokine IFN-γ, were not found in CD4 T cells of old donors in this study (Chen et al., 2003; Han et al., 2006; Mirza et al., 2011). Further studies are needed to determine the similarities and differences between mouse and human in age-associated changes in T cells.

The finding of low expression of CD28 in memory CD4 T cells from aged donors leads to further comparison of gene expression between CD28^+^ and CD28^−^ memory CD4 T cell subsets in selected old donors (65–75 years old) (Czesnikiewicz-Guzik et al., 2008). CD28 is a co-stimulatory receptor that offers a critical signal for complete T cell activation during T cell receptor initiation. Absence of the CD28 signal causes partial T cell activation or even anergy. Strikingly, it was found that the difference in gene expression between CD28^+^ and CD28^−^ memory CD4 T cells was greater than that of memory CD4 T cells between young and old subjects. CD28^−^ memory CD4 T cells express high levels of several KIR genes and some surface receptors genes (*CD70*, *CD74*, and *CCR5*), but low levels of *TCRB*, *CD27*, *IL7R*, *IL9R*, and *ICAM2*. Although loss of CD28 protein expression with aging is mainly found in CD8 T cells, this study shows that reduced expression of CD28 mRNA is also found in CD4 T cells with aging.

### Human CD8 T cells

A global comparative analysis of gene expression in human CD8 T cells isolated from young (23–27 year old) and old (65–81 year old) adults reported the identification of a total of 754 genes (505 down-regulated and 258 up-regulated) using an expression fold change more than 1.5 (Cao et al., 2010). The functions of these genes are involved in the regulation of transcription, protein phosphorylation, ubiquitination, intracellular transport, immune response, and apoptosis. Intriguingly, the down-regulated genes in CD8 T cells from old donors affect multiple stages of gene transcription: chromatin structure, transcription initiation, elongation, RNA stabilization, and protein translation and translocation; this suggests the impaired regulation of these fundamental molecular events might be responsible for the declined immune function in the elderly. In addition, genes related to the signaling pathways are also found down-regulated in CD8 T cells from old donors, such as T cell receptor signaling, IL-2 signaling, IGF-1 signaling, insulin receptor signaling, JAK/Stat signaling, MAPK/JNK signaling, PI3K/AKT signaling, Wnt/β-catenin signaling, and ERK/MAPK signaling. On the other hand, oxidative phosphorylation and apoptosis signaling are up-regulated in CD8 T cells from old donors. These alterations of gene expression provide a mechanism for the functional decline of the immune response in the elderly.

### CD28^−^ CD8 T cells

Accumulation of CD28^−^ CD8 T cells is one of the hallmarks of human aging. The CD28^−^ population is considered as aged CD8 T cells in humans (Weng et al., 2009). The study of CD28^+^ and CD28^−^ memory CD8 T cells (CD45RA^−^) from young and middle aged donors (25–45 years old) identified <100 altered genes/transcripts based on the criteria of the false discovery rate (FDR) <0.05 and fold change >2 (Fann et al., 2005). Among these 45 genes,CD28^−^ CD8 T cells highly expressed natural killer cell receptors, *GZMB*, *PRF1*, *FASLG*, *IL12A*, *IL12*, *CCL4*, *CX3CR1*, and *CMKLR1*. In contrast, they had low expression of *IL3*, *IL23A*, *IL7R*, and *IL12RB2*.

A subsequent study comparing CD28^+^ and CD28^−^ CD8 T cells from young and old donors found that gene expression patterns of CD28^−^ CD8 T cells from young and old subjects were similar (Lazuardi et al., 2009). In contrast, there were noticeable differences in gene expression of CD28^+^ CD8 T cells between young and old subjects. From the gene expression similarities, the levels from CD28^+^ CD8 T cells in old donors were located between young CD28^+^ and CD28^−^ (young and old) CD8 T cells. The highly expressed genes identified by Fann et al. (2005) in CD28^−^ CD8 T cells were also found in this study. In addition, high expression of *PIK3CD*, *MAL*, *IL6R*, *CD62L*, and *CCR7* were found in CD28^+^ CD8 T cells of young subjects whereas high expression of *GATA3*, *BIRC3*, *FAS*, *RGS1*, and *MAP3K1* were found in CD28^+^ CD8 T cells of old subjects. This study reveals that CD28^−^ CD8 T cells have a common pattern of gene expression regardless of whether they are isolated from young or old subjects. However, whether the difference in gene expression in CD28^+^ CD8 T cells between young and old subjects is reflecting the impact of aging or the heterogeneous nature (different percentages of naïve and memory T cells) of CD28^+^ CD8 T cells in young and old subjects remains to be determined.

Interestingly, similarly reduced expression of some co-stimulatory molecules including CD28, NK cell receptor, and chemokine receptors were found in both CD4 and CD8 CD28^−^ T cells with some differences in the expression of *CD40L*, *KLRD1*, *KLRG1*, and *KLRK1* (Czesnikiewicz-Guzik et al., 2008). However, loss of CD28 surface expression was only prominent in CD8 T cells with aging. Further studies will be needed to compare the functional alteration of CD4 and CD8 subsets with aging.

### Mouse CD4 and CD8 T cells

Studies in mouse T cells suggest that aging has a more profound detrimental impact on naïve compared to memory T cells (Ponnappan and Ponnappan, 2011). Gene expression comparison of naïve CD4 and CD8 T cells between young (3–4 months) and old (20 months) mice identified over 2000 age-associated genes in CD4 and CD8 T cells using a twofold change as the cutoff (Mirza et al., 2011). The functions of these genes are broad and involved in multiple cellular functions such as cell growth, cell cycle, cell death, inflammatory response, and cell trafficking. Some of those identified genes exhibited similar changes in both CD4 and CD8 T cells from old mice, such as *Anxa1*, *Ccl1*, *Ccl5*, *Ccr2*, *Il4*, *Havcr2*, and *Ltb4r1*. The enhanced expression of chemokines and chemokine receptors in aged mouse T cells was also observed in an earlier study (Mo et al., 2003). Some gene alterations are specific to either CD4 or CD8 T cells, such as *Jak3*, *Socs1*, and *Pi3kcd* in CD4, and *Penk*, *Nfc2*, and *Irak3* in CD8 T cells of old mice.

Gene expression analysis of *in vitro* stimulated naïve T cells between young and old mice further revealed some defects in T cell signaling, cytokine production, and differentiation into Th2 cells. Gata3 and c-Maf were found up-regulated post-activation in naïve aged CD4 T cells, which may be responsible for the imbalanced Th2 immune response in the elderly. *Ccl5* and *Tlt4* are up-regulated in aged CD8 T cells from pre- to post-activation, while *Ccl1*, *Ccl9*, *Il7r*, and other genes are only up-regulated post-activation. Genes such as *Tnfsf14* and *S100a9* are up-regulated in aged CD8 T cells only in pre- and 12 h post-activation. In CD4 aged T cells, *Ccl5* and *Tlr4* are up-regulated at all timepoints. *Rorc* is not differentially expressed before activation, however its expression was decreased in aged CD4 T cells compared with young ones after TCR activation. In the pattern of lower expression in the elderly at both pre- and post-activation, many genes associated with microtubules, cell cycle replication, migration, and other functions were found in both CD4 and CD8 naïve T cells. Taken together, the highly expressed and up-regulated specific cytokines, chemokines, and their receptors in aged naïve T cells indicate that naïve CD4 and CD8 T cells in old mice have a pro-inflammatory status.

## Gene Networks and Pathways Altered in Aged T Cells

A global view of gene expression profiles provides a means for examining the gene networks and signaling pathways of a defined biological process and function. Among the genes that were transcriptionally altered in aged T cells, substantial numbers of them are associated with basic cellular and molecular biological processes such as cell growth and proliferation, cell death and apoptosis, energy utilization and metabolism, and transcription regulation, which were also reported in other types of cells with aging (Kuilman et al., 2010). In Table 1, we combine the altered expressed genes from the literature based on their original selection and listed seven functional categories. We will focus on the molecular basis of three immune function-related alterations in T cells with aging in this review.

**Table 1:** **List of genes that are expressed either lower or higher in aged than in young T cells**.

Down-regulated			Up-regulated		
Genes	Subsets	Reference	Genes	Subsets	Reference
**T cell receptor signaling pathway**			**Cytokine/chemokine network**		

*AKT3*	h CD8	Cao et al. (2010)	*ACVR2A, IL15, TNFSF14*	h CD8	Cao et al. (2010)
*CD28*	h CD4N, CD8CD28-	Weng et al. (2009), Czesnikiewicz-Guzik et al. (2008)	*Bmpr1a*	m CD4N, CD8N	Mirza et al. (2011)
*CD3G*	h CD8	Cao et al. (2010)	*Ccl1, Il5, Il9, Il3, Il21*	m s-CD4N & CD8N	Mirza et al. (2011)
*DLG1*	h CD8	Cao et al. (2010)	*Ccl17, Ccl20, Ccl22, Ccl24*	m s-CD4N	Mirza et al. (2011)
*GRAP2*	h CD8	Cao et al. (2010)	*Ccl21c, Ccl8, Csf2*	m CD8N	Mirza et al. (2011)
*GRB2*	h CD8CD28-	Lazuardi et al. (2009)	*CCL3/Ccl3*	h CD4, m CD4N	Mirza et al. (2011), Chen et al. (2003)
*ITK*	h CD8	Cao et al. (2010)	*CCL4*	h CD8CD28-	Fann et al. (2005)
*LCK*	h CD8	Cao et al. (2010)	*CCL5/Ccl5*	h CD8, m CD4N, CD8N	Cao et al. (2010), Mirza et al. (2011)
*MAPK1, MAPK14*	h CD8	Cao et al. (2010)	*CCR1*	h CD4	Mo et al. (2003), Yung and Mo (2003)
*NCK1*	h CD8	Cao et al. (2010)	*CCR2/Ccr2*	h CD4, m CD4N, CD8N	Mirza et al. (2011), Mo et al. (2003)
*PDK1*	h CD8CD28-	Lazuardi et al. (2009)	*CCR3*	h CD4	Yung and Mo (2003)
*PIK3CD*	h CD8CD28-	Lazuardi et al. (2009)	*CCR4/Ccr4*	h CD4, CD4N, m CD4N, s-CD8N	Czesnikiewicz-Guzik et al. (2008), Mirza et al. (2011), Mo et al. (2003), Yung and Mo (2003)
*SOS2*	h CD8	Cao et al. (2010)	*CCR5/Ccr5*	h CD4, CD4N, m CD4N/CD8N	Czesnikiewicz-Guzik et al. (2008), Mirza et al. (2011), Mo et al. (2003), Yung and Mo (2003)
			*CCR6, CXCR2, CXCR5*	h CD4	Mo et al. (2003)
**Cytokine/chemokine network**			*CCR8*	h CD4N	Czesnikiewicz-Guzik et al. (2008), Mo et al. (2003)
*Ccl19*	m s-CD4N	Mirza et al. (2011)	*CD70, IL17RB*	h CD4N	Czesnikiewicz-Guzik et al. (2008)
*Ccl22*	m CD8N	Mirza et al. (2011)	*CLCF1*	h CD8CD28-	Lazuardi et al. (2009)
*Ccl24*	m s-CD8N	Mirza et al. (2011)	*Csd2, Cxcl11*	m s-CD4N	Mirza et al. (2011)
*Ccr9*	m CD4N, CD8N	Mirza et al. (2011)	*Csf1, Cxcl10*	m CD4N	Mirza et al. (2011)
*Cxcl1*	m CD4N	Mirza et al. (2011)	*Csf2ra, Cxcl2*	m CD8N	Mirza et al. (2011)
*Cxcl10, Cxcl12*	m CD8N	Mirza et al. (2011)	*Csf3r*	m s-CD8N	Mirza et al. (2011)
*CXCR7, CXCR9*	h CD4, CD8	Mo et al. (2003)	*CX3CR1*	h CD8CD28-	Lazuardi et al. (2009), Fann et al. (2005)
*Flt1*	m s-CD8N	Mirza et al. (2011)	*CXCL12*	h CD4	Hernandez-Lopez et al. (2010), Cane et al. (2012)
*Ifna1*	m CD8N, s-CD4N	Mirza et al. (2011)	*Cxcl9*	m CD8N, s-CD4N	Mirza et al. (2011)
*Ifna13*	m CD8N	Mirza et al. (2011)	*CXCR3/Cxcr3*	h CD4, CD8, m CD4N, CD8N	Cao et al. (2010), Mirza et al. (2011), Mo et al. (2003)
*Ifnab*	m CD4N	Mirza et al. (2011)	*CXCR4*	h CD4	Mo et al. (2003), Hernandez-Lopez et al. (2010), Cane et al. (2012)
*Il12b, Kit, Pdgfb*	m s-CD4N	Mirza et al. (2011)	*FAS, IFNGR1*	h CD8CD28-	Lazuardi et al. (2009)

**Cytokine/chemokine network**			**Cytokine/chemokine network**		

*Il12rb2*	m s-CD8N	Mirza et al. (2011)	*Ifng*, Il10, Il4	m CD4N, CD8N	Mirza et al. (2011)
*Il17a, Il6*	m s-CD8N	Mirza et al. (2011)	*Il12rb1, Il13, Il5ra*	m CD4N	Mirza et al. (2011)
*Il6ra*	m CD4N	Mirza et al. (2011)	*Il18r1, Il20ra, Il23r, Il7r*	m s-CD8N	Mirza et al. (2011)
*Kit*	m s-CD8N	Mirza et al. (2011)	*Il18rap, Il1b*	m CD8N	Mirza et al. (2011)
*Ppbp*	m CD4N	Mirza et al. (2011)	*Il19, Il24, Il2ra, Inhba*	m s-CD4N	Mirza et al. (2011)
*Tnfrsf21*	m s-CD8N	Mirza et al. (2011)	*Il1r2*	m CD4N, s-CD4N, CD8N	Mirza et al. (2011)
*Tnfsf11a*	m CD8N	Mirza et al. (2011)	*Il2, Il22, Il23a*	m s-CD8N	Mirza et al. (2011)
*Tpo*	m s-CD8N	Mirza et al. (2011)	*Ltbr*	m CD4N, CD8N	Mirza et al. (2011)
*Xcr1*	m CD4N, s-CD8N	Mirza et al. (2011)	*Tnfrsf13c*	m CD4N	Mirza et al. (2011)
**Natural killer cell-mediated cytotoxicity**			*Tnfsf13b, Tnfsf4, Tnfrsf17*	m CD4N, CD8N	Mirza et al. (2011)
*Ifna1*	m s-CD4N	Mirza et al. (2011)			
*Klrd1, Klrk1*	m s-CD4N	Mirza et al. (2011)	**Natural killer cell-mediated cytotoxicity**		
*Nfatc4*	m s-CD4N	Mirza et al. (2011)	*CD244*	h CD8	Cao et al. (2010)
			*FAS*	h CD8CD28-	Lazuardi et al. (2009)
**Toll like receptor signaling pathway**			*GZMB*	h CD8CD28-	Lazuardi et al. (2009)
*Cd86*	m CD8N	Mirza et al. (2011)	*IFNGR1*	h CD8CD28-	Lazuardi et al. (2009)
*Cxcl10*	m CD8N	Mirza et al. (2011)	*ITGB2*	h CD8	Cao et al. (2010)
*Ifna1, Ifna13*	m CD8N	Mirza et al. (2011)	*KIR2DL3, KIR2DL4*	h CD4N	Czesnikiewicz-Guzik et al. (2008)
*Ifnab*	m CD4N	Mirza et al. (2011)	*KIR2DL5A*	h CD8CD28-	Lazuardi et al. (2009)
*Il6*	m s-CD8N	Mirza et al. (2011)	*KIR2DS5*	h CD8CD28-	Lazuardi et al. (2009)
*Irf7*	m CD4N, s-CD8N	Mirza et al. (2011)	*KIR3DL1*	h CD8CD28-	Lazuardi et al. (2009)
*Lbp*	m CD4N	Mirza et al. (2011)	*KIR3DL2*	h CD4N, CD8CD28-	Czesnikiewicz-Guzik et al. (2008), Lazuardi et al. (2009)
*Pik3cd*	m CD4N	Mirza et al. (2011)	*KLRC3*	h CD8CD28-	Lazuardi et al. (2009)
*Rela*	m CD4N	Mirza et al. (2011)	*KLRD1*	h CD8	Cao et al. (2010)
*Tlr5, Tlr7*	m s-CD8N	Mirza et al. (2011)	*KLRG1*	h CD8	Henson and Akbar (2009)
			MAPK1	h CD8CD28-	Lazuardi et al. (2009)
**Cell cycle regulator**			*NKG2D*	h CD4	Alonso-Arias et al. (2011)
*CCND2, CCNH*	h CD8	Cao et al. (2010)	*PRF1*	h CD8CD28-	Lazuardi et al. (2009)
*FZR1*	h CD8	Cao et al. (2010)	*PTPN6*	h CD8	Cao et al. (2010)
*MAD1L1*	h CD8	Cao et al. (2010)	*VAV3*	h CD8CD28-	Lazuardi et al. (2009)
*MYC*	h CD8	Cao et al. (2010)	
*RAD21*	h CD8	Cao et al. (2010)	**MAPK signaling pathway**		
*RB1, RBL2*	h CD8	Cao et al. (2010)	*DUSP2, DUSP5,DUSP6, DUSP10*	h CD8	Cao et al. (2010)
*SMAD2, SMAD4*	h CD8	Cao et al. (2010)	*DUSP4*	h CD8CD28-	Lazuardi et al. (2009)
*SMC3*	h CD8	Cao et al. (2010)	*FAS*	h CD8CD28-	Lazuardi et al. (2009)
*STAG1*	h CD8	Cao et al. (2010)	*FGFR1*	h CD8	Cao et al. (2010)
*TFDP2*	h CD8	Cao et al. (2010)	*FLNA*	h CD8	Cao et al. (2010)
*YWHAE, YWHAZ*	h CD8	Cao et al. (2010)	*GNA12*	h CD8	Cao et al. (2010)
			*MAP3K1*	h CD8CD28-	Lazuardi et al. (2009)
**Insulin signaling pathway**			*MAP3K5*	h CD8	Cao et al. (2010)
*AKT3*	h CD8	Cao et al. (2010)	*MAP3K8*	h CD8	Cao et al. (2010)
*BRAF*	h CD8	Cao et al. (2010)	*MAP4K1*	h CD8	Cao et al. (2010)
*FLOT2*	h CD8	Cao et al. (2010)	*MAPK1*	h CD8CD28-	Lazuardi et al. (2009)
*FOXO1*	h CD8	Cao et al. (2010)	*NR4A1*	h CD8	Cao et al. (2010)
*GRB2*	h CD8CD28-	Lazuardi et al. (2009)	*RPS6KA5*	h CD8	Cao et al. (2010)

**Insulin signaling pathway**			**MAPK signaling pathway**		

*IRS1, IRS2*	h CD8	Cao et al. (2010)	*RRAS2*	h CD8CD28-	Lazuardi et al. (2009)
*MAPK1*	h CD8	Cao et al. (2010)			
*PDE3B*	h CD8	Cao et al. (2010)	**Toll like receptor signaling pathway**		
*PIK3CD*	h CD8CD28-	Lazuardi et al. (2009)	*Ccl3*	m CD4N	Mirza et al. (2011)
*PIK3R2*	h CD8	Cao et al. (2010)	*Ccl5*	m CD4N	Mirza et al. (2011)
*PRKACB, PRKAR1A*	h CD8	Cao et al. (2010)	*Cd86*	m CD4N	Mirza et al. (2011)
*RPS6KB1*	h CD8	Cao et al. (2010)	*Cxcl10*	m CD4N	Mirza et al. (2011)
*SOS2*	h CD8	Cao et al. (2010)	*Tlr4*	m CD4N	Mirza et al. (2011)

*h, Human; m, mouse; CD4N, CD4 naïve T cells; CD8N, CD8 naïve T cells; s-, TCR activated*.

### Reduced expression of TCR and co-stimulation signaling associated genes

**Figure 1:**
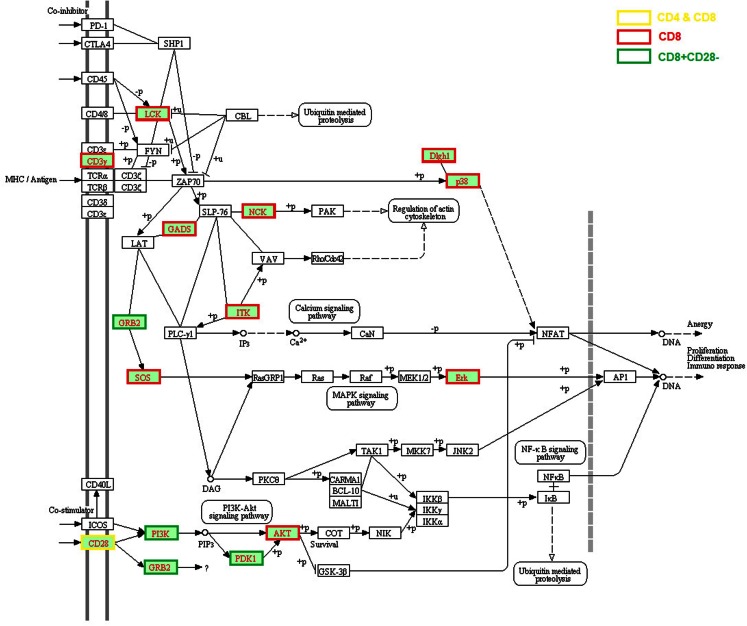
**Age-related alteration of gene expression in the TCR signaling pathway**. The age-related genes were assembled from the literature based on the significant changes reported by the original articles which were applied for all figures (Chen et al., 2003; Mo et al., 2003; Yung and Mo, 2003; Fann et al., 2005; Czesnikiewicz-Guzik et al., 2008; Henson and Akbar, 2009; Lazuardi et al., 2009; Weng et al., 2009; Cao et al., 2010; Hernandez-Lopez et al., 2010; Alonso-Arias et al., 2011; Mirza et al., 2011; Cane et al., 2012). Three-hundred five up-regulated and 472 down-regulated genes in aged human T cells including CD4 and CD8 T cells were analyzed using KEGG analysis in the WEB-based GEne SeT AnaLysis Toolkit (WebGestalt). The entire TCR signaling pathway is included and the molecules with lower gene expression compared with young are highlighted in red text and different border color indicating specific T cell subsets. The altered expressed genes in the TCR signaling pathway include *CD3G*, *LCK*, *GRAP2* (encode GADS), *GRB2*, *ITK*, *NCK*, MAPK signaling pathway molecules *SOS2* and *MAPK1* (encode Erk), *CD28*, PI3K-AKT signaling pathway molecules *PI3K*, *PDK1*, and *AKT*, DLG1 (encode DLGH1) and *MAPK14* (encode p38).

Impaired TCR signal transduction was observed in aged mice and humans two decades ago (Utsuyama et al., 1993; Whisler et al., 1996). Genes associated with several TCR signaling pathways were found to be expressed lower in aged hosts on both genome-scale (Table 1; Figure 1) and individual studies. First, *CD3G* expression was lower in human CD8 T cells (Cao et al., 2010), and phosphorylated Cd3z was decreased in aged mouse CD4 T cells, and almost absent in 18-month old mice (Garcia and Miller, 1997). Second, significantly reduced expression of CD28, particularly in CD8 T cells, was widely reported in humans with aging (Weng et al., 2009). Lower expression of CD28 was also found in human CD4 memory cells but to a much lower degree compared to CD8 T cells (Czesnikiewicz-Guzik et al., 2008). Intriguingly, several age-associated defects in TCR signaling occur at the post-translational level without obvious changes in the level of gene expression, such as: reduced phosphorylation of phospholipase Cγ1 (PLCγ1), JNK, second messengers such as inositol trisphosphate (IP3) and diacylglycerol (DAG), reduced influx of Ca2^+^ (Utsuyama et al., 1997; Kirk et al., 1999) in T cells of aged mice, decreased LCK activity (Fulop et al., 1999), and ERK phosphorylation in T cells of aged humans (Li et al., 2012). The consequence of these age-associated changes in TCR signaling is observed at the transcriptional levels of their downstream targets, such as the decreased expression of *SOS*, *GADS*, *NKC*, *ITK*, *PI3K*, *PDK2*, *AKT*, *Erk*, *Dlgh1*, and *p38* (Table 1) (Utsuyama et al., 1993; Whisler et al., 1996). Certainly, more studies are needed to dissect the primary causes of the impaired expression of these age-related genes and their functional consequences.

### Alteration of cytokines/chemokines network

**Figure 2:**
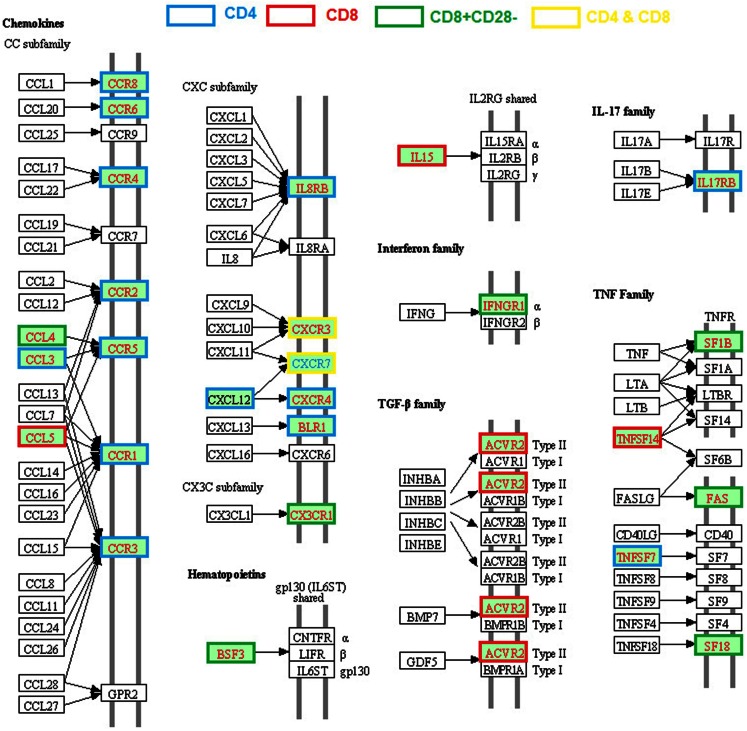
**Age-related alteration of gene expression regarding cytokine–cytokine receptor interactions**. The gene families of chemokines, interferon, TGFβ, IL17, and TNF are included and the molecules with higher or lower gene expression compared with young are highlighted in red and blue text, respectively. The different border color indicates specific T cell subsets.

Alteration of expression of various cytokines, chemokines, and their receptors in CD4 or CD8 T cells from old humans and mice are well documented (Table 1; Figure 2). Unlike the age-associated TCR signaling pathway, enhanced expression of various cytokines of the TNF and TGF-β families, chemokines (*CCL3*, *CCL4*, *CCL5*, *CXCL12*), and chemokine receptors (*CCR1*, *CCR2*, *CCR3*, *CCR4*, *CCR5*, *CCR*6, *CCR8*, *CXCR2*, *CXCR3*, *CXC4*, *CXCR5*) were reported in CD4 or CD8 T cells from old humans and mice (Mo et al., 2003; Yung and Mo, 2003; Fann et al., 2005; Lazuardi et al., 2009; Cane et al., 2012).

Age-related enhanced expression of cytokines and their receptors include *IL15*, *IL17RB*, TNF family members (*TNFSF7* and *TNFSF14*), and receptors (*FAS*, *TNFRSF18*, and *TNFSF1B*), IFN family receptor *IFNGR1*, and TGF-β family receptors *ACVR2A*. Most of these genes are involved in the inflammatory response, which could be the result of an increased inflammatory state during some chronic infections. Chemokine *CXCL12* and its receptor *CXCR4* are involved in regulating thymocyte development and differentiation from DN (CD44^−^CD25^+^, CD44^−^CD25^−^) to DP stages (Ara et al., 2003), but their roles in aged T cells have not been determined. In general, the high levels of expression of chemokines/chemokine receptors lead to an enhanced T cell chemotactic response and different patterns of tissue migration/residency of T cells in the old subjects (Cane et al., 2012). It is unclear whether such changes are a result of an increased inflammatory state and/or some chronic infection. Regardless, it is apparent this age-related change in T cell chemokine expression has an important functional consequence.

### Enhanced gene expression related to NK cells function

**Figure 3:**
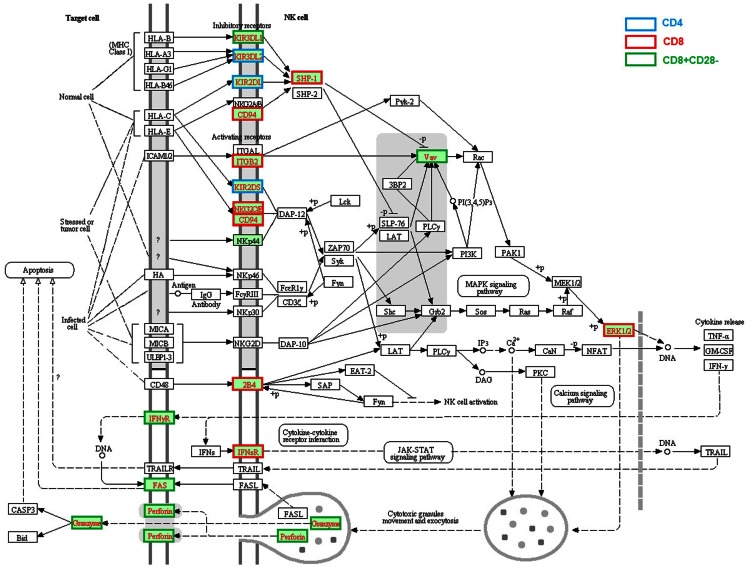
**Age-related alteration of gene expression regarding NK cell-mediated cytotoxicity**. NK cell-mediated cytotoxicity was present and the molecules with higher gene expression compared with young were highlighted in red text. The different border color indicates specific T cell subsets. The altered expressed genes associated with NK mediated cytotoxicity in aged T cells include receptors *KIR3DL1*, *KIR3DL2*, *KIR2DL*, *KIR2DS*, *KLRD1* (encode CD94), *ITGB2*, *NKG2C/E*, *CD244* (encode 2B4), *IFNGR*, *IFNSR*, and *FAS*, signaling molecules *PTPN6* (*SHP-1*), *VAV* and *MARK1* (ERK1), and effector molecules *GZMB* and *PRF1* (encode Perforin).

Enhanced expression of a cluster of genes associated with NK cell function is one of the most profound age-related changes in T cells, especially in human CD28^−^ CD8 T cells (Table 1; Figure 3); it includes killer cell lectin-like receptors (*KLR*, *KLRC3*, *KLRD1*, and *KLRG1*), killer cell immunoglobulin-like receptors (*KIR2DL3*, *KIR2DL4*, *KIRDL5A*, *KIR2DS5*, *KIR3DL1*, and *KIR3DL2*), and *CD244*. Although these genes were primarily expressed in NK cells, aged memory, or CD28^−^ T cells appear to increase their expression. Since CD28^−^ cells are a unique subset of T cells in older humans, the acquired expression of NK cell receptors and some cytotoxic molecules (*GZMB* and *PRF1*) might reflect chronic infection and an increased inflammatory state (Fann et al., 2005). Another possibility is that gaining expression of NK receptors, especially KIRs, might be a compensation for a shrinking TCR repertoire in aged hosts (Abedin et al., 2005; Vallejo, 2006) since KIR recognizes MHC-I/Ag complex with lower affinity compared with that of TCR and was found only in the later stage of proliferative lifespan of oligoclonal T cells (Snyder et al., 2004). If this is true, aged T cells with a high level of NK cell receptors can function in both an MHC-restricted and non-restricted manner.

## Conclusion

In the past decade, we have learned enormously from the global gene expression analysis of aged T cells. Alteration of several gene networks and pathways that are associated with aged T cells have now been identified in humans and mice, including T cell receptor and activation-related molecules, alteration of chemokine/chemokine receptor expression, gain of NK cell receptors and functions, and etc. Whether these identified alterations of gene expressions occur in all cells or in subsets of defined T cell populations remains to be determined. It is sufficient to say these alterations contribute to the overall decline of T cell function.

It is worth mentioning that not all microarray data, especially for some of the early studies, have the highest quality. The shortcomings include: incomplete gene list of the array used, insufficient number of biological repeats, and lack of standardized selection criteria of significantly altered genes. It is necessary in future studies to use the whole genome microarray chips with better designed experiments, such as using defined cell populations, standardized stimulation conditions and time, sufficient numbers of the biological repeats, and proper statistical tools for selection of significantly altered genes. In addition, future studies will need to dissect which specific stage of T cell development and differentiation these changes occurred. The precise relationship of gene expression change and function is essential for a better understanding these age-associated alterations in T cell functions.

The current commercial whole genome microarray chips offered by Agilent, Affymetrix, and Illumina have greatly improved from a few years ago, and become a standard method for global gene expression analysis. However, some shortcomings are also emerging with recent developments. Most of the microarray chips for gene expression analysis do not include miRNA and lncRNA, both of which serve critical biological functions and in which some alterations with aging and cell differentiation were recently reviewed (Gorospe and Abdelmohsen, 2011; Guttman et al., 2011). These microarray chips also do not provide information on different splicing isoforms of transcribed genes; thus, global transcriptional assessment by the traditional microarray method does not offer complete transcriptional information. RNA-Seq, a relatively new method based on next-generation sequencing, can readily address these shortcomings and provide more complete transcriptional analysis.

Identification of the alteration of several gene networks and pathways that are associated with aged T cells paves a way for further functional assessment and potential clinical interventions. Although the general approach such as supplementation of Vitamin E in mice showed some promising results, such as increased expression of genes involved in cell cycle regulation (*Ccnb2*, *Cdc2*, and *Cdc6*) and higher up-regulation of expression in old T cells following stimulation, a more focused approach targeting specific genes and gene networks that are altered in aged T cells may offer a more direct and specific treatment. With the advancement of the tools that can be used in manipulating gene expression in cells, experimental determination and verification of these specific genes/gene networks based therapeutic approach is feasible and will lead to the development of novel drugs that could postpone or even reverse the changes associated with aging in T cells.

## Conflict of Interest Statement

The authors declare that the research was conducted in the absence of any commercial or financial relationships that could be construed as a potential conflict of interest.
